# The role of hormone dosages in the assessment of cervical lesions by FNA: A descriptive analysis of 762 cases^✰^

**DOI:** 10.1016/j.clinsp.2024.100539

**Published:** 2024-11-28

**Authors:** Arthur Henrique Cunha-Volpato, Carlos Izaias Sartorão Neto, Luiz Fernando Ferraz da Silva, Paulo Campos Carneiro

**Affiliations:** a Diagcel Laboratory and Rede D'OR - São Luiz Pathology Laboratory, São Paulo, SP, Brazil; b Faculdade de Medicina da Universidade de São Paulo, São Paulo, SP, Brazil; c Department of Pathology, Faculdade de Medicina da Universidade de São Paulo, São Paulo, SP, Brazil; d Managing Partner of Lab. Diagcel, São Paulo, SP, Brazil

**Keywords:** Fine-needle aspiration, Needle washout hormone measurement, Thyroglobulin, Calcitonin, Parathormone, Cutoff value

## Abstract

**Introduction:**

The use of Hormone Dosage (HD) in Fine Needle Aspiration Biopsy (FNA) needle washout fluid, introduced in the 1990s, initially aimed at measuring Thyroglobulin (TG). Elevated TG concentration in an extrathyroidal cervical lesion suggests metastases of Well-Differentiated Thyroid Carcinomas (WDTC). Over the years, HD has evolved to incorporate Calcitonin (CT) and Parathormone (PTH), improving sensitivity and specificity in diverse clinical scenarios. Despite varying cutoffs in the literature, existing data underscore significant advancements in diagnostic accuracy.

**Methods:**

This paper presents a case series of 762 cervical lesions with FNA cytology and HD in different cervical regions.

**Results:**

It was possible to establish the histogenesis of the lesion based on a combination of the FNA cytology and PTH HD findings, so PTH was useful to determine parathyroid origin in all regions, Thyroglobulin (TG) and PTH together provide insights on cell origin in difficult cases. Thyroglobulin (TG) dosage enhances the accuracy of detecting well-differentiated thyroid carcinomas. In post-thyroidectomy by bed lesions, dosages help to determine non-thyroid/parathyroid lesions.

**Discussion:**

The case series and review underscore the valuable contribution of HD in fine-needle aspiration for the evaluation of cervical lesions. By integrating HD with cytological, ultrasound, and clinical data, clinicians can enhance the accuracy and specificity of FNA in diagnosing various cervical lesions, ultimately improving patient care and management.

## Highlights

• Hormone measurement in FNA washout fluid is an accessible method for improving accuracy.
• Thyroglobulin (TG) dosage can enhance the accuracy of detecting well-differentiated thyroid carcinomas.
• Calcitonin dosage complements cytology in detecting medullary thyroid carcinoma cervical metastasis.
• Post-thyroidectoby bed dosages help to determine non-thyroid/parathyroid lesions.

## Introduction

The concept of employing Thyroglobulin (TG) Hormone Dosage (HD) in FNA needle washout fluid for detecting Well-Differentiated Thyroid Carcinomas (WDTC) metastasis was introduced by Pacini and colleagues in 1992.^1^, ^2^, ^3^ As the years progressed, HD has been refined to incorporate CT for detecting primary or metastatic medullary carcinoma^4^, ^5^, ^6^, ^7^, ^8^, ^9^, ^10^ and PTH to differentiate thyroid and parathyroid lesions.^6^^,^^11^, ^12^, ^13^, ^14^

In today's landscape, HD is widely regarded as an accessible technique for enhancing the specificity and sensitivity of FNA in investigating cervical lesions in specific contexts. Even though the cutoff and reference values for the hormones utilized in the method are not universally agreed upon in scientific literature, existing data suggest that substantial improvements in sensitivity and specificity are achievable in various clinical scenarios.^3^^,^^8^^,^^12^^,^^15^^,^^16^

TG is a protein produced by thyroid follicular cells. Elevated serum levels are often observed in individuals with WDTC. There's no universally accepted reference value for TG in FNA washout fluid, with figures ranging from 2.3 ng/mL to 55 ng/mL in published studies.^3^^,^^15^^,^^17^, ^18^, ^19^, ^20^ The inclusion of hormone dosage in conjunction with cytological aspects can significantly increase diagnostic accuracy for WDTC cervical metastases.^15^^,^^16^^,^^18^^,^^20^^,^^21^

CT, a hormone produced by the parafollicular cells of the thyroid, can be quantified in the FNA washout fluid. Increased CT levels have been reported in Medullary Thyroid Carcinoma (MTC).^4^, ^5^, ^6^, ^7^, ^8^, ^9^, ^10^ Similarly, the dosage of PTH has been utilized to identify parathyroid nodules and to distinguish them from nodules of other origins located in the central neck compartment.^6^^,^^11^, ^12^, ^13^, ^14^^,^^22^ Both techniques, bolstered by international guidelines, play crucial roles in advancing the diagnostic approach to cervical nodules.

Presently, while HD is widely recognized as an accessible method for increasing the specificity and sensitivity of FNAs for cervical lesions, its underuse in routine diagnosis is notable, possibly due to uncertainties about its specific indications and its distinct advantages in selected cases and the absence of an integrated framework for decision making based on clinical and image data. To advocate for a broader application of HD, the authors present a comprehensive series of FNAs with HD. This series aims to offer a foundational framework for categorizing cases and formulating strategies for the judicious selection and interpretation of HD across diverse cervical lesion groups. Through this endeavor, the authors aspire to foster a wider acceptance of this technique, paving the way for a better diagnostic approach to cervical nodules.

## Materials and methods

This is a retrospective report of a series of FNA cases of cervical lesions with HD and a brief review of the literature on HD in FNA washout fluid. The reported cases were conducted at the Diagcel Laboratory, São Paulo, SP, Brazil, and the University of São Paulo Medical School. The Institutional Research Ethics Committee granted approval for this study (CAAE 62,842,422.0.0000.0068).

A total of 762 ultrasound-guided FNA cytology reports of cervical lesions were reviewed, and performed between 2015 and 2021, which had available HD results for TG, PTH, and/or CT in the FNA washout fluid. The washout fluid was obtained by rinsing the FNA needle with 1.0 mL of saline solution (NaCl 0.9 %). The needles were gently rinsed after the smears were made and the acquired fluid was directed to HD, while any residual material was preserved in formaldehyde for cell block preparation. The choice to direct part of the FNA material for HD was either determined during the collection (by the FNA-performing physician) or prior to the procedure (following the guidance of the attending physician). Notably, the decision to collect the washout fluid for HD must precede the complete cytological analysis, as material preserved in formaldehyde or alcohol is not apt for HD.

The data from the FNA was anonymized and recorded electronically. To ensure patient confidentiality, each FNA received a random code, with all identifiable details removed. Recorded variables encompassed the lesion's location, its sonographic features, HD results, and its final diagnosis. HD values either above or below the detection limits (subject to laboratory variation) were equated to these thresholds for representation in the graphs, with the inclusion of a minor random value to minimize overplotting (graphical jitter).

**Fig. 1:**
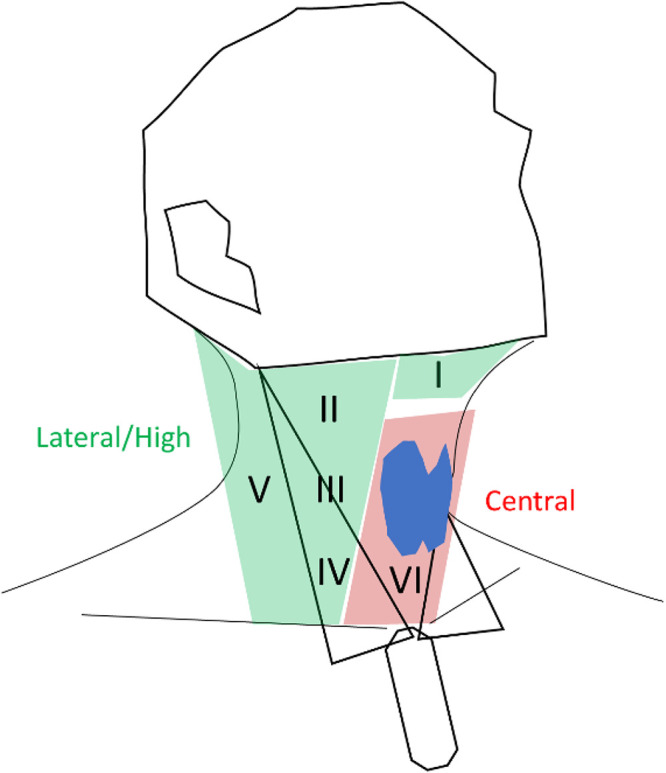
Regional divisions of the neck.

Cervical lesion locations were classified into six cervical levels.^23^ Levels I through V were deemed as lateral localizations, while level VI, encompassing thyroid lesions, was labeled as central localization (Fig. 1) and further subclassified according to the lesion relative position to the thyroid (adjacent/behind the thyroid, thyroid bed-post-thyroidectomy, thyroid-posterior/polar and thyroid-non-posterior/polar). Furthermore, cervical lesions underwent stratification as predominantly cystic or non-cystic, based on ultrasound descriptions and the FNA cytological outcomes. Cystic lesions were characterized by a dominant cystic ultrasound appearance. All other lesions were categorized as non-cystic.

Given the exclusion of a correlation between procured results and histopathological findings (gold standard), two predefined cutoff values from existing literature were utilized for graphical representation and result presentation. Given the absence of a consensus regarding the optimal cutoff for each hormone, the results are showcased as a case distribution across two cutoff levels, promoting a holistic interpretation. In our practice, the authors interpret results above the lower cutoff as moderately suspicious and, above the second cutoff, as very suspicious. As the final cytological diagnosis and the results of HD are not independent variables, it is not possible to use correlation measures between the two and it is not appropriate to calculate the sample size for adequate power.

The lesions' final diagnosis was rendered by the overseeing pathologist, factoring in the cytological findings (smears and cellblocks), clinical data, ultrasound findings, immunocytochemical examinations (when conducted), and HD outcomes. It's pertinent to note that all the participating pathologists responsible for diagnostic determination (3 in total) boast over two decades of expertise in cervical lesion cytology assessment.

## Results

The characterization of the 762 patients in terms of the location of the lesions and the percentage of cystic lesions, as well as the number of cases that underwent each of the hormone dosages, or their combinations, is shown in Table 1.

**Table 1:** Number of lesions with hormone dosage reported percentage of predominantly cystic lesions and distribution by location.

**Location**	**Lesions**				**Endocrine Markers**							
	**Total**		**Mostly Cystic**		**TG**		**PTH**		**TG + PTH**		**CT**	
Central	399	52.4 %	37	31.3 %	346	45.4 %	334	43.8 %	292	38.3 %	26	3.4 %
Thyroid	139	18.2 %	7	11.9 %	108	14.2 %	130	17.1 %	108	14.2 %	19	2.5 %
Posterior/polar	112	14.7 %	5	4.5 %	91	11.9 %	111	14.6 %	91	11.9 %	7	0.9 %
Other regions	27	3.5 %	2	7.4 %	17	2.2 %	19	2.5 %	17	2.2 %	12	1.6 %
Adjacent/Behind thyroid	185	24.3 %	26	14.1 %	165	21.7 %	162	21.3 %	142	18.6 %	2	0.3 %
Thyroid bed, post-thyroidectomy	75	9.8 %	4	5.3 %	73	9.6 %	42	5.5 %	42	5.5 %	5	0.7 %
Lateral	363	47.6 %	9	10.1 %	356	46.7 %	7	0.9 %	7	0.9 %	17	2.2 %
Cervical level I/II	87	11.4 %	2	2.3 %	86	11.3 %	0	0.0 %	0	0.0 %	3	0.4 %
Cervical level III/IV	241	31.6 %	5	2.1 %	235	30.8 %	7	0.9 %	7	0.9 %	14	1.8 %
Cervical level V	35	4.6 %	2	5.7 %	35	4.6 %	0	0.0 %	0	0.0 %	0	0.0 %
Total	762	100 %	46	6.0 %	702	92.1 %	341	44.8 %	299	39.2 %	43	5.6 %

**Fig. 2:**
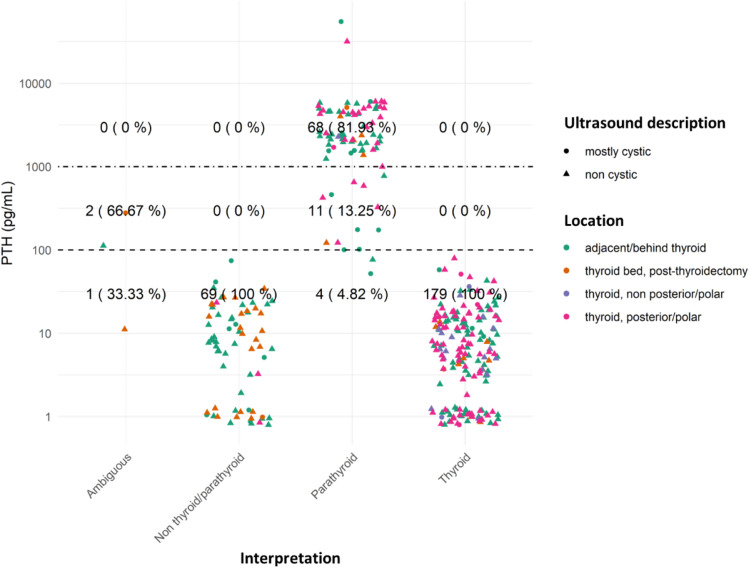
PTH dosage and interpretation of centrally located lesions. Dotted lines demarcate the applied cutoffs: > 100 pg/mL HD positive for parathyroid tissue (as per Ketha et al.)^14^ ; > 1000 pg/mL is the value typically seen in parathyroid lesions. To enhance graphical clarity and minimize dot overlap, slight random values (jittering) were introduced.

Fig. 2 visually represents centrally located lesions with PTH dosage organized according to their definitive diagnosis. The shapes identify the ultrasound profile of the lesion and colors denoting the location.

**Fig. 3:**
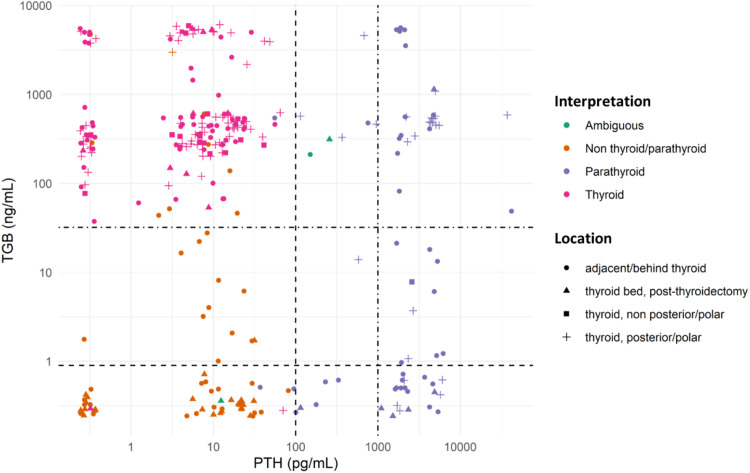
TG and PTH concentrations and interpretation of central lesions. Dotted lines demarcate the applied cutoffs: > 100 pg/mL HD positive for parathyroid tissue (as per Ketha et al.)^14^ ; > 1000 pg/mL is the value typically seen in parathyroid lesions; TG > 0.9 ng/mL is the threshold for thyroidectomized patients, and TG > 32.04 ng/mL for non-thyroidectomized patients (as per Pak et al., 2015).^20^ Although, the thyroidectomy status data isn't available for all cases. To enhance graphical clarity and minimize dot overlap, slight random values (jittering) were introduced.

The combination of TG and PTH was employed in a significant subset (299 cases), proving instrumental in differentiating these cellular types. Fig. 3 illustrates the dispersion of these cases by correlating the TG and PTH measurements and categorizing them by histogenesis defined by the final FNA cytology interpretation and FNA location.

**Fig. 4:**
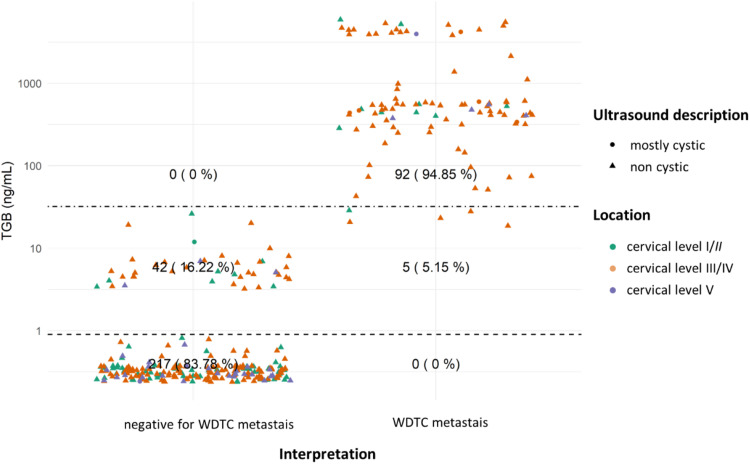
TG dosages and interpretation for lateral lesions. Dotted lines showcase both cutoffs: TG > 0.9 ng/mL is the threshold for thyroidectomized patients, and TG > 32.04 ng/mL for non-thyroidectomized patients (as per Pak et al., 2015).^20^ Although, the thyroidectomy status data isn't available for all cases. To enhance graphical clarity and minimize dot overlap, slight random values (jittering) were introduced.

**Fig. 5:**
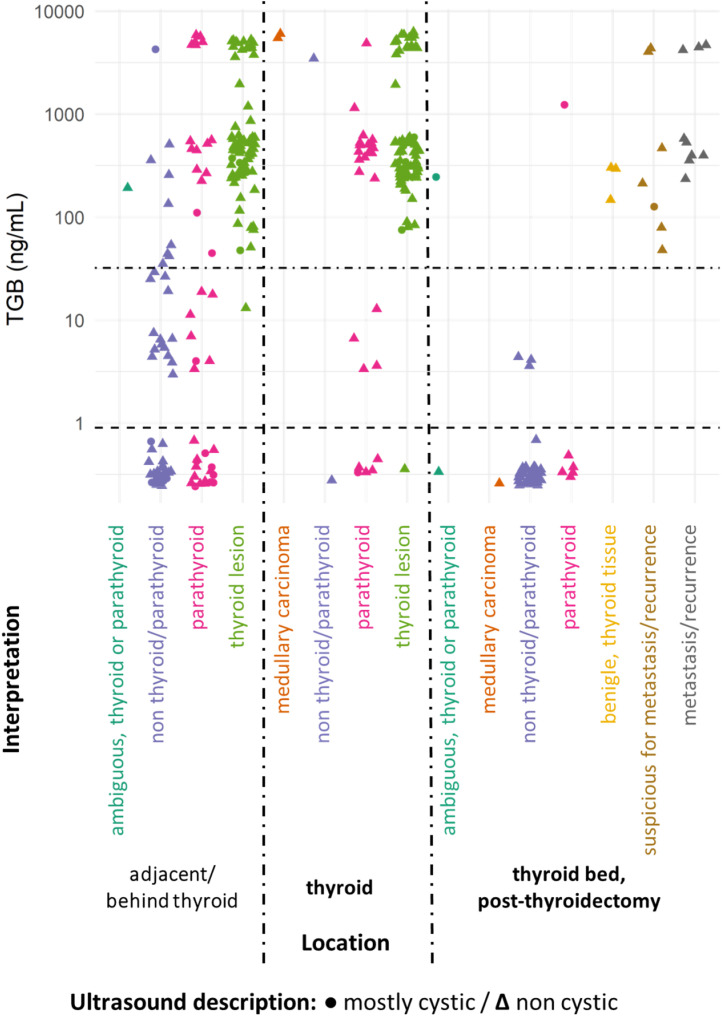
TG dosages and interpretation of centrally located lesions. Dotted lines indicate the applied cutoffs: TG > 0.9 ng/mL is the threshold for thyroidectomized patients, and TG > 32.04 ng/mL for non-thyroidectomized patients (as per Pak et al., 2015).^20^ Although, the thyroidectomy status data isn't available for all cases. To enhance graphical clarity and minimize dot overlap, slight random values (jittering) were introduced.

In evaluating TG, a primary marker for thyroid lesions (and WDTC metastases), lesions with TG dosage were organized according to their definitive diagnosis and location. This was further characterized by ultrasound features (shape) and location, as depicted in Figs. 4 and 5.

**Fig. 6:**
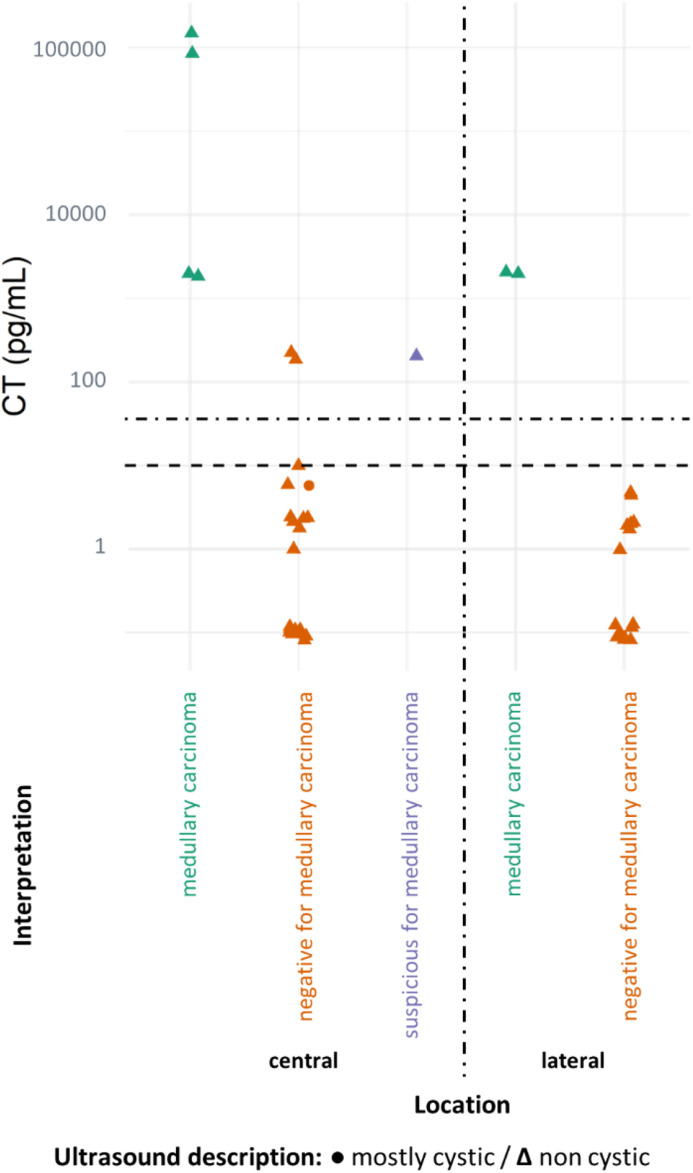
CT dosage and interpretation for both central and lateral lesions. Dotted lines indicate the chosen cutoffs: CT 〈 10 pg/mL as negative and CT 〉 36 pg/mL as positive (as per Tromboli et al., 2017).^6^ To enhance graphical clarity and minimize dot overlap, slight random values (jittering) were introduced.

Subsequently, CT HDs are showcased in relation to the medullary carcinoma diagnosis, categorized by ultrasound characteristics and lesion location in Fig. 6.

## Discussion

**Fig. 7:**
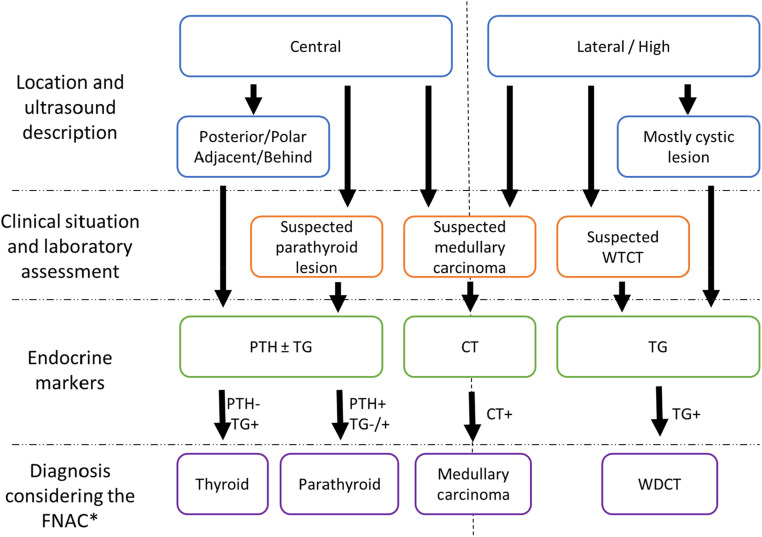
Flowchart summarizing the process for selecting and interpreting HD in cervical FNA needle washout fluids. It is essential to underscore that diagnosis invariably relies on the correlation with cytology, image, and clinical data.

In this study, the authors present a series of 762 cervical lesions with HD, characterizing the distribution of these cases in relation to hormone levels, location, and diagnostic specifics, whether in isolation or combined. The present study demonstrates that the measurement of Thyroglobulin (TG), Parathyroid Hormone (PTH), and Calcitonin (CT) in washout fluid offers valuable insights that complement cytological analysis, enhancing diagnostic accuracy and guiding clinical decision-making by providing valuable insights into the nature of cervical lesions. The decision to collect material for hormone measurement occurs during or before the FNA procedure. Therefore, is essential for all professionals involved, especially those performing the FNA and those requesting the test, to be well-acquainted with the situations in which hormone measurements contribute to diagnostic reasoning. Moreover, to prevent misinterpretation, the results of hormone measurement should be considered in conjunction with other clinical, cytological, and ultrasound data for the final cytological diagnosis. A flowchart summarizing the decision-making process (Fig. 7) can serve as a valuable tool in guiding the selection and interpretation of hormone measurements in FNA.

PTH hormone dosage helps distinguish between thyroid and parathyroid tissue, which can be challenging relying solely on cytology, as shown by the low sensitivity of FNA isolated in this identification^24^ The posterior/polar regions of the thyroid and adjacent/posterior to the thyroid (regions in which the parathyroid glands are often located) concentrate the requests for PTH HD. In most of the cases analyzed, except for three, which were considered ambiguous, it was possible to establish the histogenesis of the lesion based on a combination of the FNA cytology and PTH HD findings (Figs. 2 and 3). These observations align with those of Florentino and colleagues^12^ who showed that the combination of FNA with PTH HD in the washout fluids yields a 93.7 % sensitivity and 100 % specificity in identifying parathyroid tissue.

Thyroglobulin, in turn, increases the sensitivity of the FNA cytology to detect metastases of WDTC, especially in laterally located lesions. Lateral lesions with high TG levels are at least suspicious for WDTC metastases, even when they are hypocellular (which is often the case with cystic lesions). There are reports of benign thyroid tissue in lymph nodes in the lateral cervical region,^25^ which is a possibility to be considered when a thyroid lesion suspicious for WDTC is not identified. However, the authors considered this a remote possibility when compared with the most likely scenario of an occult thyroid carcinoma.

Extensive studies have highlighted the variability in the diagnostic accuracy of TG measurement in the FNA to detect WDTC metastasis. Sensitivity and specificity are largely dependent on the selected cutoff for Thyroglobulin (TG) dosage and lesion location, as demonstrated by various research findings. For instance, Xiao and colleagues^15^ observed a significant increase in sensitivity, from 81 % to 91 %, with the combination of FNA cytology + TG compared to FNA cytology alone. This improvement is further corroborated by a meta-analysis conducted by Zhu and colleagues^18^ which revealed high sensitivity (91 %) and specificity (94 %) for TG measurements in FNA washout fluid. Notably, specificity can be enhanced even further by higher cutoffs, such as 40 ng/mL. On the contrary, Song, and colleagues,^26^ in their analysis of FNA exclusively of lymph nodes in the central compartment of the neck (cervical level VI), found no significant differences in diagnostic accuracy between FNA cytology + TG and FNA cytology alone. These results emphasize the critical importance of considering the cutoff, lesion location, and the methodology employed in TG measurement when assessing sensitivity and specificity, as those factors can significantly impact the diagnostic accuracy of the technique.

Several studies have also investigated the correlation between TG levels in FNA washout fluids and serum thyroglobulin levels. Sun and colleagues^17^ reported no significant correlation between the two values. Conversely, Kim and colleagues^27^ found an increase in diagnostic specificity, albeit with lower sensitivity, when using a cutoff for the ratio of FNA TG to serum TG, as compared to using a cutoff to the FNA TG. J. Wang and colleagues^21^ identified no relationship between serum TG and FNATG dosage in cases with low serum TG or in thyroidectomized patients, however, a significant correlation was observed between the two values in cases with high serum TG levels or non-thyroidectomized patients. It's important to note that the accuracy of FNATG dosage did not significantly differ between thyroidectomized and non-thyroidectomized patients in their study.^21^

The interpretation of FNA TG levels in the central neck lesions should be conducted with caution, as even momentary passage of the needle through the thyroid can lead to elevated TG values. This phenomenon was observed in our cases: many centrally located lesions had elevated FNA TG levels, irrespective of the final FNA diagnosis (Figs. 3 and 5). Additionally, certain benign extrathyroidal lesions may contain thyroid tissue, such as thyroglossal duct, ectopic thyroid tissue, and exophytic thyroid nodules. Those observations align with the findings of Song and colleagues,^26^ who noted that, for lymph nodes in the central compartment of the neck, the Positive Predictive Value (PPV) for Papillary Thyroid Carcinoma (PTC) metastasis based on TG dosage in FNA washout fluid (87.5 %) was lower than the PPV of FNA cytology alone (92.6 %) or cytology combined with TG (88.5 %), when a cutoff of 14.6 ng/mL was used. Furthermore, in post-thyroidectomy scenarios, elevated TG levels may not definitively indicate WDTC metastasis or recurrence due to the potential presence of residual benign thyroid tissue.

It's imperative to consider that less differentiated tumors can yield false-negative hormone measurements, potentially affecting the method's sensitivity.^28^, ^29^, ^30^ In such instances, a comprehensive evaluation that combines clinical data, FNA cytology, FNA HD, and ultrasound findings is essential for an accurate diagnosis.

Calcitonin emerges as a pivotal biomarker for distinguishing medullary carcinoma from other thyroid tumors and identifying metastases or recurrences. This is particularly relevant for post-thyroidectomy patients with biochemical recurrence but lacking suspicious lesions on ultrasound. Multiple studies have consistently demonstrated a substantial increase in sensitivity for diagnosing MTC when calcitonin measurements are incorporated into FNA cytology. For example, Trimboli and colleagues^10^ reported an FNA sensitivity increase from 54 % to 95 % with CT dosage in FNA washout fluid. Marques and colleagues^8^ also showed an increase in sensitivity from 82 % to 100 % and in specificity from 98 % to 99.9 %. Liu and colleagues reported a sensitivity increase from 55 % to 98 % in the diagnosis of MTC micronodules (≤ 10 mm).^9^ However, it is important to emphasize that the sensitivity of FNA CT measurements can vary depending on the type of lesion and the presence of less differentiated metastases. Therefore, FNA cytology and ultrasound findings should be considered in conjunction with FNA CT for an accurate diagnosis.

Interestingly, in the present sample, there were two centrally located cases with elevated FNA CT that were ultimately considered negative for MTC (Fig. 6). One of these cases was interpreted as a parathyroid lesion (with concomitantly elevated FNA PTH) and the other as PTC (with low FNA CT levels in cervical metastases). This finding underscores the importance of considering both HD and FNA cytology results in the final interpretation.

Although this series provides valuable insights into the applicability and interpretation of hormone measurements in FNA, it is essential to acknowledge its limitations. The lack of correlation between FNA and hormone measurements with the histopathological results impedes the calculation of diagnostic sensitivity and specificity. Additionally, it is worth noting the potential impact of variations in hormone measurement methodologies across different clinical laboratories employed in our cases, albeit with similar characteristics. Nevertheless, the primary aim was to discuss the contribution of these tests to the interpretation of FNA cytology, which constitutes a significant contribution to the existing literature.

## Conclusion

In conclusion, this case series review underscore the valuable contribution of Hormone Dosage (HD) in fine Fine-Needle Aspiration (FNA) for the evaluation of cervical lesions. By integrating HD with cytological, ultrasound, and clinical data, clinicians can enhance the accuracy and specificity of FNA in diagnosing various cervical lesions, ultimately improving patient care and management.

## Authors’ contributions

Cunha-Volpato: Investigation (Diagnostic Determination), Data Curation and Data analysis and Writing-Original Draft, Sartorão Neto: Methodology and Data Collection and Analysis; Silva: Conception, Methodology, Investigation (Diagnostic Determination), Writing-Manuscript Revision, Carneiro: Conception, Methodology, Investigation (Diagnostic Determination), Writing-Manuscript Revision.

## Funding

This research did not receive any specific grant from funding agencies in the public, commercial, or not-for-profit sectors.

## Declaration of competing interest

The authors declare no conflicts of interest.
